# Supplementary material for: Synthetic Conditions, Physical Properties, and Antibacterial Activities of Silver Nanoparticles with Exopolysaccharides of a Medicinal Fungus
Source: Materials (Basel). 2022 Aug 16;15(16):5620. doi: 10.3390/ma15165620 (PMC9412466; doi:10.3390/ma15165620)
Supplement: Supplementary file 1 [file materials-15-05620-s001.zip › materials-1818380-supplementary.pdf]

Supplementary Materials

"Yang and Wu: Synthetic conditions, physical properties and antibacterial activities of silver nanoparticles with exopolysaccharides of a medicinal fungus"

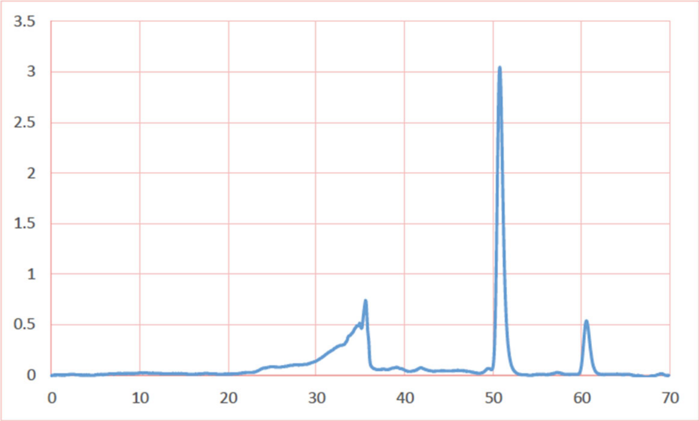

Figure S1. HPGPC spectrum for the molecular weight distribution of EPS-1.

Table S1. Molecular weight distribution of EPS-1.

Retention time (min)	Molecular weight (Da)	% Area
35.622	649883	28.72
50.831	222	60.37
60.663	60.663	10.92

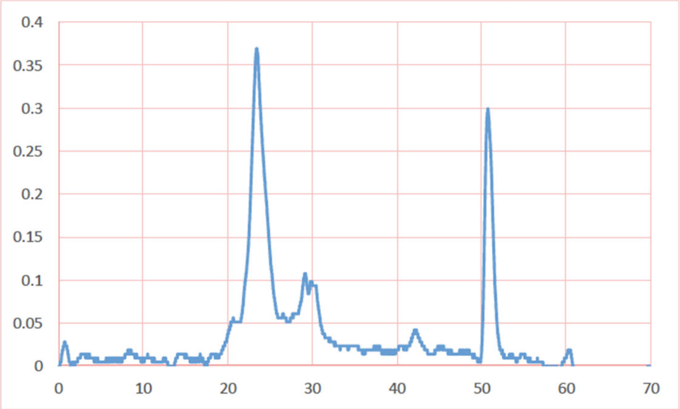

Figure S2. HPGPC spectrum for the molecular weight distribution of EPS-2

Table S2. Molecular weight distribution of EPS-2

Retention time (min)	Molecular weight (Da)	% Area
23.450	386036950	56.92
29.283	18083149	8.33
50.817	224	29.36
60.633	60.633	2.59

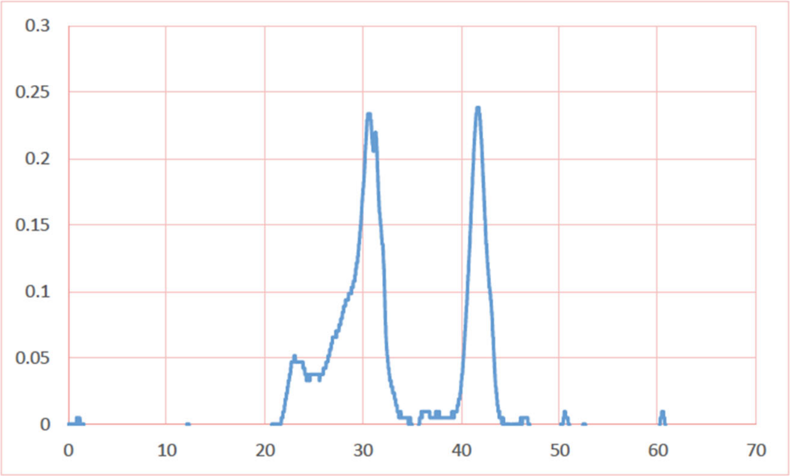

Figure S3. HPGPC spectrum for the molecular weight distribution of EPS-3

Table S3. Molecular weight distribution of EPS-3

Retention time (min)	Molecular weight (Da)	% Area
30.567	9221806	65.14
41.9	24103	34.86

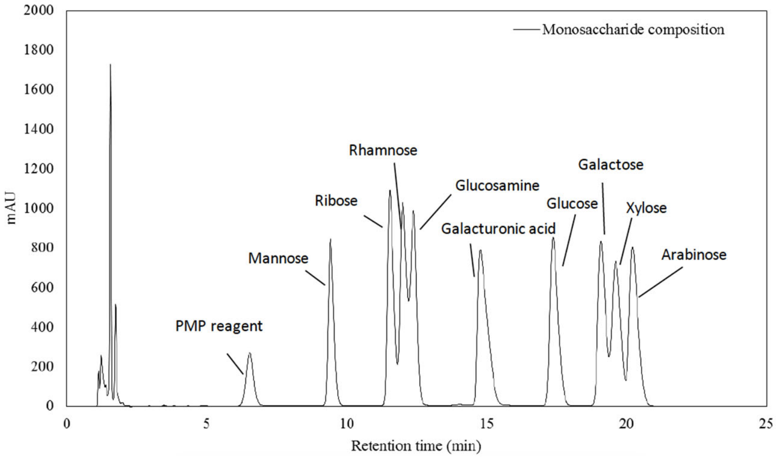

Figure S4. HPLC of monosaccharide standards.

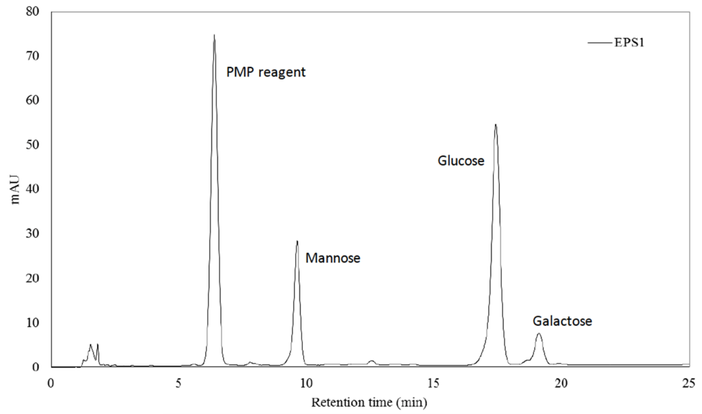

Figure S5. HPLC analysis of monosaccharide composition of EPS-1.

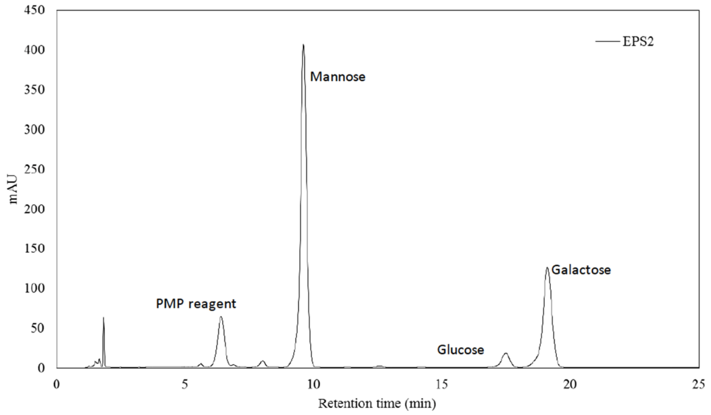

Figure S6. HPLC analysis of monosaccharide composition of EPS-2.

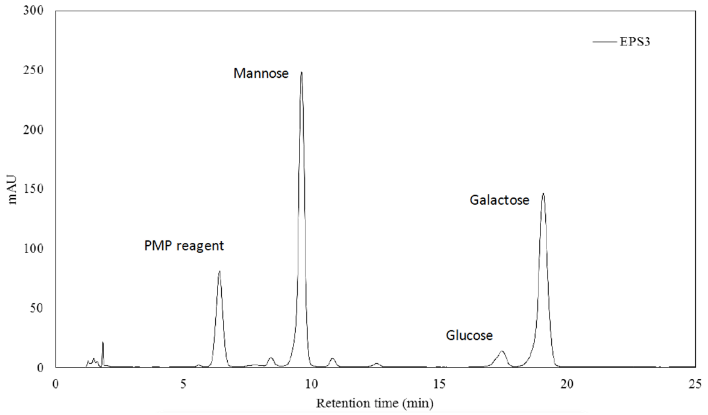

Figure S7. HPLC analysis of monosaccharide composition of EPS-3.

(a)

β-D-Galf
1
↓
3
{[6)-β-D-Glcp-(1→4)-α-D-Manp-(1→4)-α-D-Glcp-(1]$_2$→6)-α-D-Glcp-(1→[3)-α-D-Manp-(1→4)-α-D-Glcp-(1]$_2$→)$_n$

(b)

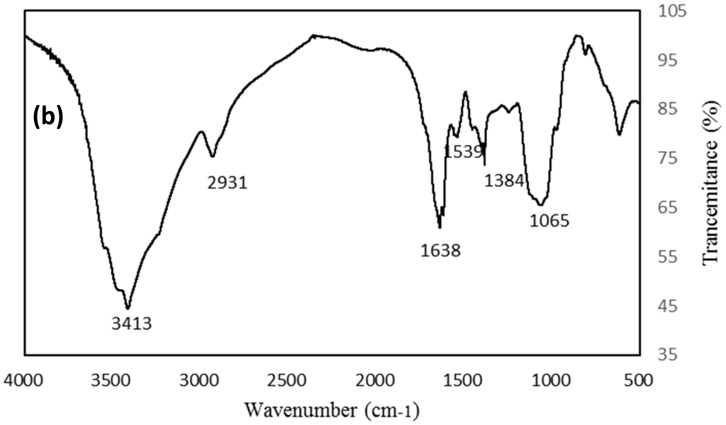

(c)

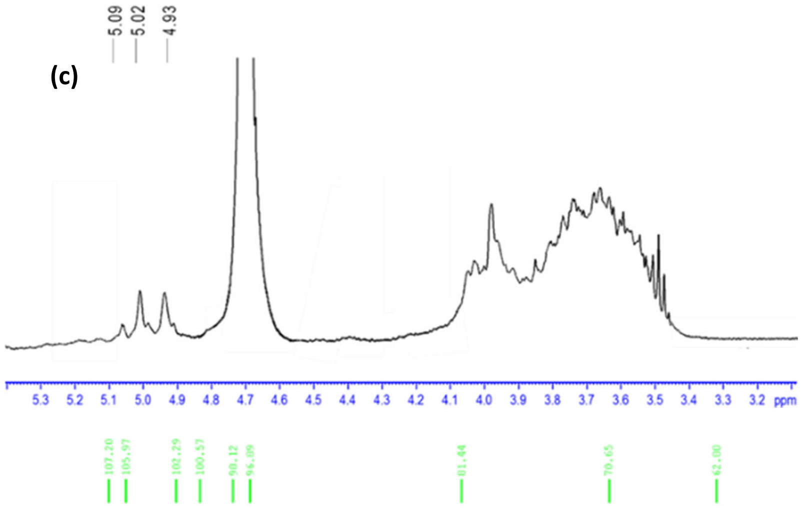

(d)

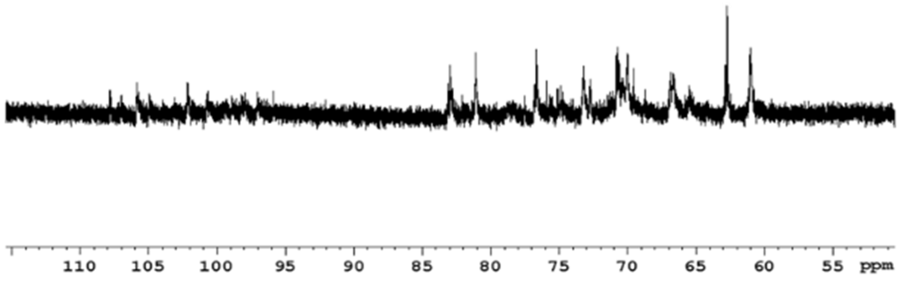

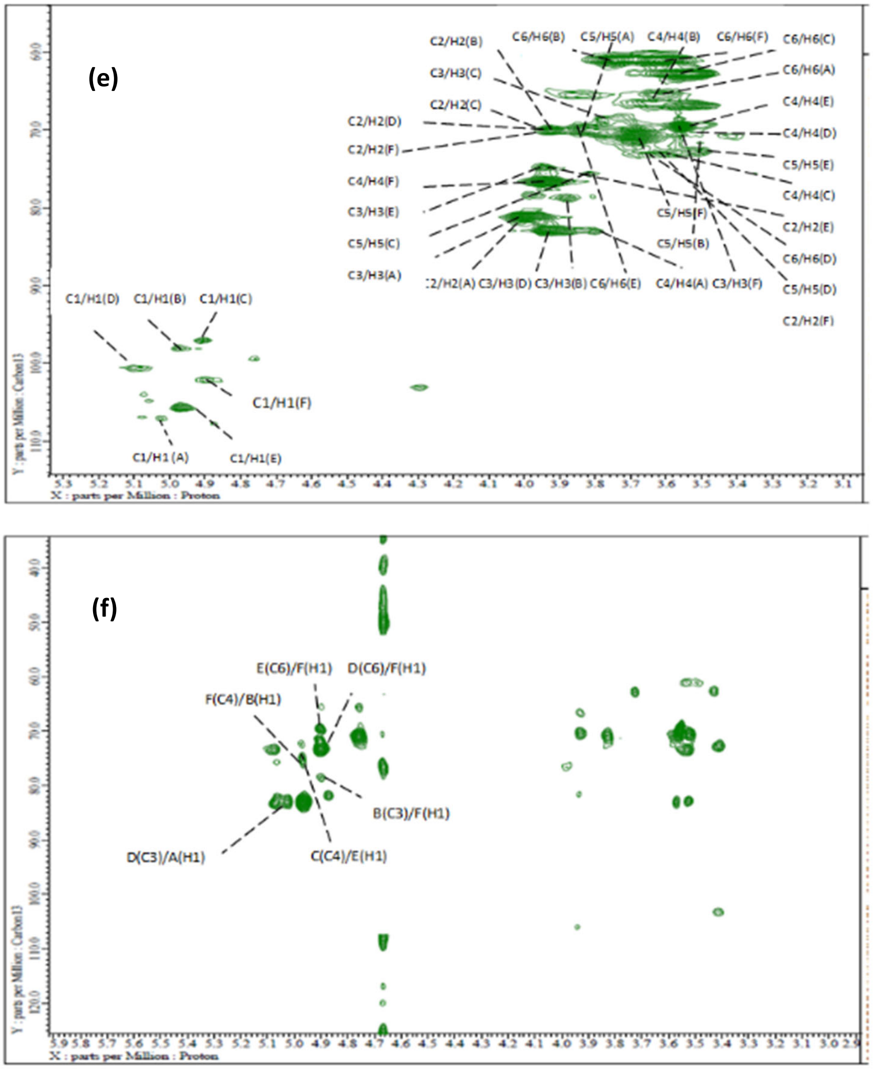

Figure S8 Structure analysis and characterization of EPS-3: (a) Repeating structure unit of an EPS fraction; (b) FT-IR spectrum; (c) ^{1}H NMR spectrum; (d) ^{13}C NMR spectrum; (e) HSQC NMR spectrum (2D); (f) HMBC spectrum (2D).

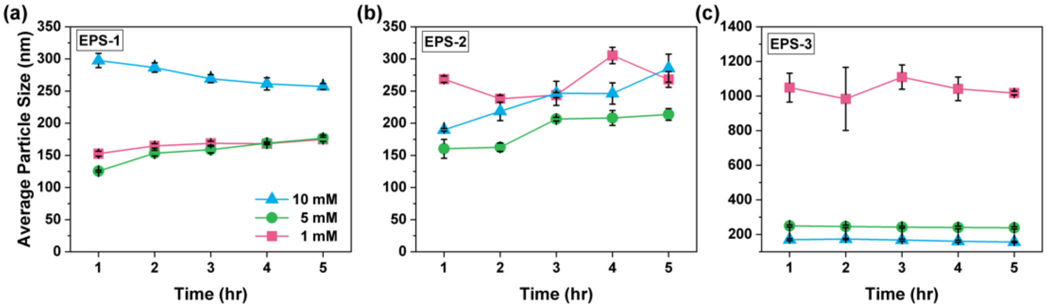

Figure S9. Average particle size of Ag NPs synthesized with 0.5 g/L EPS and various concentration of $AgNO_3$ (1, 5 and 10 mM) for (a) EPS-1, (b) EPS-2 and (c) EPS-3 as a function of reaction time. All the above experiments were conducted in the dark at 100 °C.

Table S4. The particle size comparison of Ag NPs synthesized with different EPS before and after dialysis.

EPS-Ag NPs	Particle hydro-diameter (nm)	
	Before dialysis	After dialysis
EPS-1 Ag NPs	158.9 ± 5.1	241.5 ± 3.3
EPS-2 Ag NPs	245.0 ± 17.0	430.0 ± 22.5
EPS-3 Ag NPs	206.4 ± 3.6	397.8 ± 8.9
